# Supplementary material for: Severity of bovine tuberculosis is associated with innate immune-biased transcriptional signatures of whole blood in early weeks after experimental Mycobacterium bovis infection
Source: PLoS One. 2020 Nov 9;15(11):e0239938. doi: 10.1371/journal.pone.0239938 (PMC7652326; doi:10.1371/journal.pone.0239938)
Supplement: S1 Table — (DOCX) [file pone.0239938.s007.docx]

**Table S1. Mean lesion scores and lymph node weights (g) with 95% confidence intervals**

|  | Severe Lesion Group | Moderate Lesion Group |
| --- | --- | --- |
| Total LN Score | 6.0 [6.0,6.0] | 2.3 [-0.54,5.4] |
| Total Lung Score | 25.0 [25,25] | 17.3 [9.7,24.9] |
| Mediastinal LN (wt) | 234.5 [75.9,393] | 73.0 [-111.0,256.9] |
| Tracheobronchial LN (wt) | 58.8 [20.6,97.0] | 26.7 [-21.4,74.7] |
