# Supplementary material for: Severity of bovine tuberculosis is associated with innate immune-biased transcriptional signatures of whole blood in early weeks after experimental Mycobacterium bovis infection
Source: PLoS One. 2020 Nov 9;15(11):e0239938. doi: 10.1371/journal.pone.0239938 (PMC7652326; doi:10.1371/journal.pone.0239938)
Supplement: S2 Table — Cell type enrichment scores were obtained from lists of differentially expressed genes. Scored > 2 were considered significant. AvB10down = enriched in severe compared to moderate at 10 wpi; AvB10up = enriched in moderate compared to severe at 10 wpi; AvB4down = enriched in severe compared to moderate at 4 wpi; AvB4up = enriched in moderate compared to severe at 4 wpi; BvC10down = enriched in control compared to severe at 10 wpi; BvC10up = enriched in severe compared to control at 10 wpi; BvC4down = enriched in control compared to severe at 4 wpi; BvC4up = enriched in severe compared to control at 4 wpi. (DOCX) [file pone.0239938.s008.docx]

**Table S2. Cell type enrichment scores from comparisons of uninfected, moderately-affected, and severely-affected cattle.** Cell type enrichment scores were obtained from lists of differentially expressed genes. Scored > 2 were considered significant. AvB10down = enriched in severe compared to moderate at 10 wpi; AvB10up = enriched in moderate compared to severe at 10 wpi; AvB4down = enriched in severe compared to moderate at 4 wpi; AvB4up = enriched in moderate compared to severe at 4 wpi; BvC10down = enriched in control compared to severe at 10 wpi; BvC10up = enriched in severe compared to control at 10 wpi; BvC4down = enriched in control compared to severe at 4 wpi; BvC4up = enriched in severe compared to control at 4 wpi.

| **Cells** | **AvB10down** | **AvB10up** | **AvB4down** | **AvB4up** | **AvC4up** | **BvC10down** | **BvC10up** | **BvC4down** | **BvC4up** |
| --- | --- | --- | --- | --- | --- | --- | --- | --- | --- |
| 3T3-L1 | 0.690519206 | 1.359107331 | 1.143026905 | 1.027527791 | 0.195042841 | 0.504041987 | 0.579589907 | 0.02054228 | 0.290260872 |
| B Cells (GL7 neg; KLH) | 0.585564032 | 0.011275117 | 1.275388128 | 0.034350937 | 0 | 0.190975227 | 0.543804776 | 0.339849318 | 0.115171869 |
| B Cells (GL7 pos; Alum) | 0.712677015 | 0.026705888 | 0.687230314 | 0.031551023 | 0 | 0.30562274 | 0.6042632 | 0.386603753 | 0.132972031 |
| B Cells (GL7 pos; KLH) | 0.647412288 | 0.022484491 | 0.642092263 | 0.063634258 | 0 | 0.19441041 | 0.521265829 | 0.326158342 | 0.13722173 |
| B Cells (GL7 neg; Alum) | 0.823525473 | 0.005135734 | 1.015081431 | 0.018771457 | 0 | 0.058370369 | 0.638563631 | 0.210443368 | 0.170377155 |
| B Cells Marginal Zone | 1.10309077 | 0.001735673 | 1.381908088 | 0.022115681 | 0 | 0.151292702 | 0.864895522 | 0.256496992 | 0.097432158 |
| BA/F3 | 1.261089952 | 0.015843052 | 0.377733616 | 0.006365988 | 0.246060212 | 0.133839926 | 1.395282621 | 0.094931521 | 0.110671271 |
| C2C12 | 0.329667245 | 1.137802831 | 0.897309034 | 0.628049836 | 0 | 0.161665957 | 0.276315923 | 0.091774878 | 0.095681061 |
| Stem Cells C3H/10T1/2 | 0.294547563 | 1.06421806 | 0.307457809 | 0.765289358 | 0 | 0.098764385 | 0.232048486 | 0.005936602 | 0.054910024 |
| Embryonic Fibroblasts | 0.277073094 | 1.070746855 | 0.43268759 | 0.264051305 | 0 | 0.189060366 | 0.311930768 | 0.015761605 | 0.209493245 |
| NK Cells | 0.18697012 | 0.312951372 | 0.090259431 | 0.011247913 | 0 | 1.78754146 | 0.041644343 | 0.553808284 | 0.002774111 |
| RAW 264.7 Cells | 2.047873378 | 0.040295159 | 0.163511118 | 0.060101542 | 0.628569894 | 0.037668157 | 5.435009009 | 0.014015313 | 0.515848407 |
| CD4+ T cells | 9.92E-05 | 0.260935138 | 0.002334166 | 0.048558878 | 0 | 2.095144956 | 3.38E-06 | 0.61662771 | 0.000336116 |
| CD8+ T cells | 0.002420361 | 1.218793015 | 0.014799555 | 0.04805735 | 0 | 3.478448975 | 4.76E-05 | 0.609956383 | 0.000322814 |
| Foxp3+ Tcells | 0.256967128 | 0.224930536 | 0.103987559 | 0.003432222 | 0 | 1.062094759 | 0.005879829 | 0.614206122 | 0.02622597 |
| Adipose Brown | 0.197283446 | 0.001430032 | 0.346894625 | 0.114034372 | 0.347187175 | 0.000473647 | 0.213453315 | 0.000117652 | 0.529639566 |
| Adipose White | 0.53974679 | 0.306538221 | 0.298134061 | 0.221607669 | 0.113825851 | 0.023280917 | 0.040547166 | 0.002108805 | 0.00199617 |
| Adrenal Gland | 0.139727823 | 0.877202321 | 0.235892471 | 0.043640632 | 0.143582199 | 0.237103187 | 0.001454554 | 0.021926079 | 0.004112625 |
| Amygdala | 4.24E-05 | 1.277711395 | 0.001542807 | 0.089226881 | 0.620246073 | 0.281417662 | 4.49E-08 | 2.43E-05 | 1.32E-11 |
| Bladder | 0.789826935 | 1.002775662 | 0.060178976 | 0.472929807 | 0.10631768 | 0.165323107 | 0.078208198 | 0.014551404 | 0.074895368 |
| Bone | 2.855173509 | 0.018315287 | 0.350701754 | 0.991742418 | 0 | 0.002748832 | 2.358058527 | 0.02400176 | 0.329789948 |
| Bone Marrow | 2.631898953 | 0.006756196 | 0.212615677 | 2.672944384 | 0 | 0.027400487 | 2.806891633 | 0.158356947 | 0.398118466 |
| Cerebellum | 9.38E-05 | 4.000034628 | 0.008153437 | 0.114672827 | 0.879942308 | 1.347179874 | 3.77E-06 | 0.011310579 | 5.01E-08 |
| Cerebral Cortex | 1.10E-05 | 2.12315317 | 0.001896749 | 0.070227428 | 0.637461107 | 0.439688893 | 9.83E-08 | 7.63E-05 | 2.15E-10 |
| Cerebral Cortex Prefrontal | 3.47E-06 | 2.009245645 | 0.001090882 | 0.071121947 | 0.592834976 | 0.285357652 | 5.25E-09 | 4.50E-05 | 6.97E-11 |
| Ciliary Bodies | 0.034339998 | 0.544802326 | 0.007961951 | 0.441455893 | 0.776330718 | 0.027400487 | 4.31E-05 | 0.006807038 | 8.06E-07 |
| Common Myeloid Progenitor | 0.347602043 | 0.081406199 | 0.026507645 | 0.296337827 | 0 | 0.24178515 | 0.181000003 | 0.760219985 | 0.009720404 |
| Cornea | 0.05731198 | 0.130383435 | 0.014392815 | 0.139533542 | 0.169428275 | 0.004257234 | 0.003274816 | 0.000429352 | 2.15E-05 |
| CD8a+ Dend. Cells Lymphoid | 4.264546403 | 0.006920421 | 1.756100507 | 0.459006788 | 0.254589251 | 0.020653477 | 3.399921532 | 0.277055032 | 1.386880877 |
| CD8a+ Dend. Cells Myeloid | 10.33428931 | 0.229486688 | 2.933308045 | 1.075544495 | 0 | 0.325028866 | 8.767538857 | 0.61007151 | 3.23927527 |
| B220+ Dend. Cells | 2.166227251 | 0.00190915 | 0.602681998 | 0.056296398 | 0 | 0.034363442 | 1.380557695 | 0.120714035 | 1.091408475 |
| Dorsal Root Ganglia | 0.01323299 | 0.86261618 | 0.037147439 | 0.154399829 | 0.806222806 | 0.279187704 | 0.000970782 | 0.000178074 | 0.000325879 |
| Dorsal Striatum | 2.85E-05 | 1.784098948 | 0.000415684 | 0.04253224 | 0.678426648 | 0.316495367 | 4.55E-09 | 0.000102782 | 1.34E-11 |
| Embryonic Stem Line Bruce4 P13 | 0.003155319 | 0.003376547 | 0.030552171 | 0.006548532 | 0 | 0.008147388 | 5.47E-05 | 0.000103125 | 3.75E-06 |
| Embryonic Stem Line V26 2 P16 | 7.24E-05 | 0.002211949 | 0.008805908 | 0.012204297 | 0 | 0.001277606 | 1.05E-06 | 6.38E-06 | 9.82E-07 |
| Epidermis | 0.003016321 | 0.003696397 | 0.013239468 | 0.073185403 | 0.723406452 | 0.000517788 | 8.22E-05 | 2.99E-06 | 2.07E-05 |
| Eyecup | 0.269423244 | 0.127533056 | 0.014220264 | 0.4566221 | 0.423998783 | 0.003727637 | 0.05257184 | 0.011069499 | 0.008931065 |
| Follicular B Cells | 1.240130869 | 0.050654866 | 0.897311046 | 0.013462543 | 0 | 0.89780833 | 0.789775557 | 0.451035936 | 0.13479387 |
| Granulo Monoprogenitor | 0.91719564 | 0.040986917 | 0.088995871 | 0.319851219 | 0.349908577 | 0.138790419 | 0.725324791 | 0.202793963 | 0.117359077 |
| Granulocytes | 2.354785297 | 0.038361747 | 0.134345299 | 3.063318298 | 0 | 0.141376703 | 2.456452524 | 0.305831868 | 0.311231907 |
| Heart | 0.189700146 | 0.341697862 | 0.031500087 | 0.153022168 | 0.41856613 | 0.114871634 | 0.320445894 | 0.000731917 | 0.348726665 |
| Hippocampus | 3.23E-05 | 2.532157204 | 0.00202156 | 0.133430337 | 0.72606677 | 0.474984844 | 8.27E-08 | 0.000414299 | 1.18E-08 |
| Hypothalamus | 0.000474444 | 3.333855324 | 0.00826554 | 0.280379824 | 0.384525808 | 0.79989301 | 1.18E-08 | 0.003900289 | 1.86E-11 |
| Large Intestine | 0.308566681 | 0.017724346 | 0.040294761 | 0.082102826 | 0.09413731 | 0.01138058 | 0.152424574 | 0.000366199 | 0.003601851 |
| Small Intestine | 2.111355476 | 0.05370251 | 1.09335643 | 0.198368837 | 0.624235545 | 0.058441201 | 1.629472798 | 0.001407445 | 0.384763988 |
| Iris | 0.061851664 | 0.261465586 | 0.008970819 | 0.253341381 | 0.791090959 | 0.023077879 | 0.000132062 | 0.005784415 | 0.000100984 |
| Kidney | 0.30887545 | 0.20823221 | 0.389202391 | 0.121152762 | 0.080958123 | 0.023461642 | 0.098705062 | 4.78E-05 | 0.037125035 |
| Lacrimal Gland | 0.0289823 | 0.617302973 | 0.006114991 | 0.175344368 | 0.181885779 | 0.104391411 | 0.004202898 | 0.003703451 | 0.003155614 |
| Lens | 0.025953535 | 0.331098464 | 0.090948115 | 1.077372162 | 0.537265079 | 0.012110193 | 0.000996502 | 0.005167443 | 0.002450049 |
| Liver | 0.329154031 | 5.79E-06 | 0.405659962 | 0.007771299 | 1.050783698 | 0.000760463 | 0.111759833 | 7.92E-06 | 0.187404894 |
| Lung | 1.654911423 | 0.107982594 | 0.225347327 | 0.45224454 | 0.26131132 | 0.007203828 | 1.204604791 | 0.001182193 | 0.08525793 |
| Lymph Nodes | 3.71054052 | 0.339625071 | 1.644363972 | 0.0228809 | 0 | 1.108929302 | 2.237688794 | 0.115887795 | 0.374071661 |
| mIMCD-3 Cells | 0.435183587 | 0.016091864 | 1.003731113 | 0.171889382 | 0 | 0.033758241 | 0.222302779 | 1.85E-05 | 0.035140766 |
| Macrophage Bone Marrow | 8.669740018 | 0.021352778 | 2.678521179 | 0.299068418 | 0.504693519 | 0.010040297 | 15.39859751 | 0.010143763 | 5.463171361 |
| Macrophage Bone Marrow Lps 24 Hrs | 17.4313256 | 0.03986801 | 4.8253955 | 0.55440338 | 0.983575284 | 0.023891981 | 25.61800383 | 0.022123338 | 14.3327884 |
| Macrophage Bone Marrow Lps 2Hrs | 13.72260943 | 0.015469085 | 4.1131862 | 0.644468313 | 0.155405363 | 0.003182069 | 23.15519755 | 0.029877436 | 10.26083456 |
| Macrophage Bone Marrow Lps 6Hrs | 22.10252039 | 0.000731455 | 8.795981384 | 0.466975623 | 0.934527183 | 0.000147139 | 34.97350404 | 0.002519545 | 23.14539861 |
| Macrophage Peri | 7.862108392 | 0.005436922 | 0.741141499 | 0.048571629 | 0.602350676 | 0.117094239 | 10.74238447 | 0.047905779 | 4.376784431 |
| Macrophage Peri Lps 1Hrs | 11.90410294 | 0.0027099 | 1.923182271 | 0.061892851 | 1.066940399 | 0.038988833 | 17.59188432 | 0.011841386 | 8.093015194 |
| Macrophage Peri Lps 7Hrs | 24.27130164 | 0.00010674 | 9.519134915 | 0.00321861 | 1.451517541 | 5.70E-05 | 41.11036589 | 7.04E-05 | 27.29903969 |
| Mammary Gland | 0.64049663 | 0.316962114 | 0.383246595 | 0.649509733 | 0.175892078 | 0.059954881 | 0.606875046 | 0.01190797 | 0.184911282 |
| Mammary Gland Non-Lactating | 0.328138367 | 0.766444027 | 0.070672374 | 0.528501382 | 0.367930914 | 0.078183652 | 0.035407601 | 0.004558049 | 0.040917401 |
| Mast Cells | 0.467703691 | 0.202743535 | 0.014909 | 1.219195979 | 0.170839966 | 0.715117012 | 2.631504577 | 0.918294656 | 0.912702902 |
| Mast Cells IgE | 0.453053638 | 0.448549977 | 0.02754914 | 1.310001579 | 0.198404561 | 0.362335149 | 0.729109777 | 1.065211685 | 0.059938917 |
| Mast Cells IgE 1hr | 0.453397317 | 0.074512635 | 0.08518683 | 0.775208273 | 0.626327527 | 0.423043838 | 1.810382273 | 0.608894348 | 0.590651277 |
| Mast Cells IgE 6hr | 0.346996729 | 0.243048933 | 0.028150533 | 0.657150494 | 0 | 0.725734209 | 1.101834547 | 0.920521523 | 0.254493679 |
| Megaerythrocyte Progenitor | 0.033381487 | 0.000696196 | 0.037869882 | 0.002289336 | 0 | 0.001085889 | 0.16361026 | 0.043158081 | 0.06661923 |
| Microglia | 13.13733512 | 0.043346032 | 3.442697649 | 0.196152703 | 0.718489925 | 0.054203633 | 14.66755369 | 0.002622091 | 6.699494787 |
| MIN6 cells | 0.000334286 | 0.376441915 | 0.000142154 | 0.043440304 | 0.122651728 | 0.012402617 | 1.93E-08 | 0.000565766 | 3.10E-10 |
| Neuro2a Neuroblastoma Cells | 0.008050139 | 0.475376397 | 0.094248973 | 0.748317316 | 0.148302239 | 0.684557393 | 0.006052776 | 0.013216992 | 0.000890744 |
| NIH 3T3 | 0.522039999 | 0.263379398 | 0.551717005 | 0.097315002 | 0.207572768 | 0.090041015 | 0.145366058 | 0.005366469 | 0.115255152 |
| Nucleus Accumbens | 4.71E-06 | 1.7539044 | 7.47E-05 | 0.048414159 | 0.348025777 | 0.586564644 | 1.97E-09 | 0.00136945 | 1.37E-12 |
| Olfactory Bulb | 1.26E-06 | 2.960794612 | 0.000206741 | 0.175369323 | 0.396086541 | 0.648033763 | 1.76E-09 | 0.008135795 | 1.28E-10 |
| Osteoblast Day14 | 0.110011956 | 1.360940639 | 0.249061279 | 0.177845752 | 0.105290454 | 0.295785863 | 0.094712773 | 0.00902564 | 0.017401273 |
| Osteoblast Day21 | 0.416060922 | 1.329021946 | 0.241229497 | 0.337802828 | 0.103870632 | 0.278709197 | 0.491179441 | 0.01708761 | 0.099081762 |
| Osteoblast Day5 | 0.339546275 | 1.076310229 | 0.190269576 | 0.155872901 | 0.131989799 | 0.245539805 | 0.144726875 | 0.010051674 | 0.04280382 |
| Osteoclasts | 11.42796446 | 0.069065132 | 3.507862341 | 0.728145934 | 0.185364848 | 0.085141286 | 16.22745711 | 0.061235715 | 6.888224995 |
| Ovary | 0.718449582 | 1.825164956 | 0.441684529 | 0.480518233 | 0.136275208 | 0.108570488 | 0.061767839 | 0.020222745 | 0.011395438 |
| Pancreas | 0.003911496 | 0.115900692 | 0.011888715 | 0.173820257 | 0 | 0.003240679 | 3.51E-07 | 0.057905736 | 0.000102486 |
| Pituitary | 0.001753312 | 1.77149659 | 0.014456966 | 0.340338956 | 0.425370037 | 0.643299774 | 6.42E-06 | 0.004682279 | 2.60E-06 |
| Placenta | 1.513844655 | 0.731946265 | 0.386173907 | 0.645398177 | 0.744827282 | 0.191331065 | 0.453237919 | 0.086178253 | 0.141992013 |
| Prostate | 0.114944834 | 0.021034837 | 0.101342126 | 0.025147834 | 0.125845032 | 0.000591128 | 0.000927282 | 0.001388135 | 0.002962436 |
| Retina | 1.29E-06 | 0.465570877 | 8.62E-05 | 0.228579512 | 0.502319267 | 0.001422649 | 5.27E-11 | 7.79E-05 | 1.57E-11 |
| Retinal Pigment Epithelium | 0.010755769 | 0.965612425 | 0.004901082 | 0.172931251 | 0.104274136 | 0.017368204 | 1.76E-05 | 0.002134571 | 1.95E-07 |
| Salivary Gland | 0.035158722 | 0.386040401 | 0.001569949 | 0.119854618 | 0.188126319 | 0.093613437 | 4.22E-05 | 0.003089052 | 5.41E-06 |
| Skeletal Muscle | 0.111982555 | 0.662371601 | 0.007563212 | 0.560607373 | 0.376148859 | 0.194885321 | 0.04366796 | 0.006044741 | 0.066572217 |
| Spinal Cord | 2.22E-06 | 2.40683751 | 0.002376403 | 0.202022785 | 0.411559952 | 0.764011561 | 6.84E-08 | 0.013022127 | 1.05E-07 |
| Spleen | 3.35171289 | 0.538037764 | 1.16965 | 0.464581983 | 0 | 1.126614006 | 1.725244331 | 0.719975888 | 0.4846709 |
| Hematopoietic Stem Cells | 0.154533466 | 0.611591063 | 0.057462376 | 0.99195103 | 0 | 0.56539841 | 0.132512344 | 1.360641687 | 0.002673986 |
| Stomach | 0.10142367 | 0.000233425 | 0.026434443 | 0.072178324 | 0.942611398 | 0.000174047 | 0.004286191 | 8.48E-06 | 0.000212146 |
| Testis | 2.57E-12 | 0.008301607 | 1.30E-06 | 5.57E-05 | 0.035531496 | 1.10E-07 | 0 | 3.42E-14 | 0 |
| CD4+/CD8+ DP Thymocytes | 0.000914146 | 0.171686028 | 0.031755897 | 0.028658065 | 0 | 0.961613674 | 1.70E-06 | 0.21119145 | 0.000103001 |
| CD4+ SP Thymoctyes | 0.035065147 | 0.514067849 | 0.013300219 | 0.015416885 | 0 | 1.506890266 | 0.000732432 | 0.661386403 | 0.012316408 |
| CD8+ SP Thymocytes | 0.002373488 | 0.737748308 | 0.01464249 | 0.004288026 | 0 | 1.830926931 | 0.000142398 | 0.862383722 | 0.000697738 |
| Umbilical Cord | 0.078350776 | 0.262873003 | 0.001055062 | 0.640917269 | 0.328782489 | 0.014844089 | 0.001141887 | 0.002596658 | 0.000125334 |
| Uterus | 2.082789024 | 1.453501388 | 0.27790864 | 1.324564127 | 0.415212712 | 0.281366998 | 0.986281002 | 0.03899 | 0.16095187 |
