# Supplementary figures and images for: Severity of bovine tuberculosis is associated with innate immune-biased transcriptional signatures of whole blood in early weeks after experimental Mycobacterium bovis infection
Source: PLoS One. 2020 Nov 9;15(11):e0239938. doi: 10.1371/journal.pone.0239938 (PMC7652326; doi:10.1371/journal.pone.0239938)

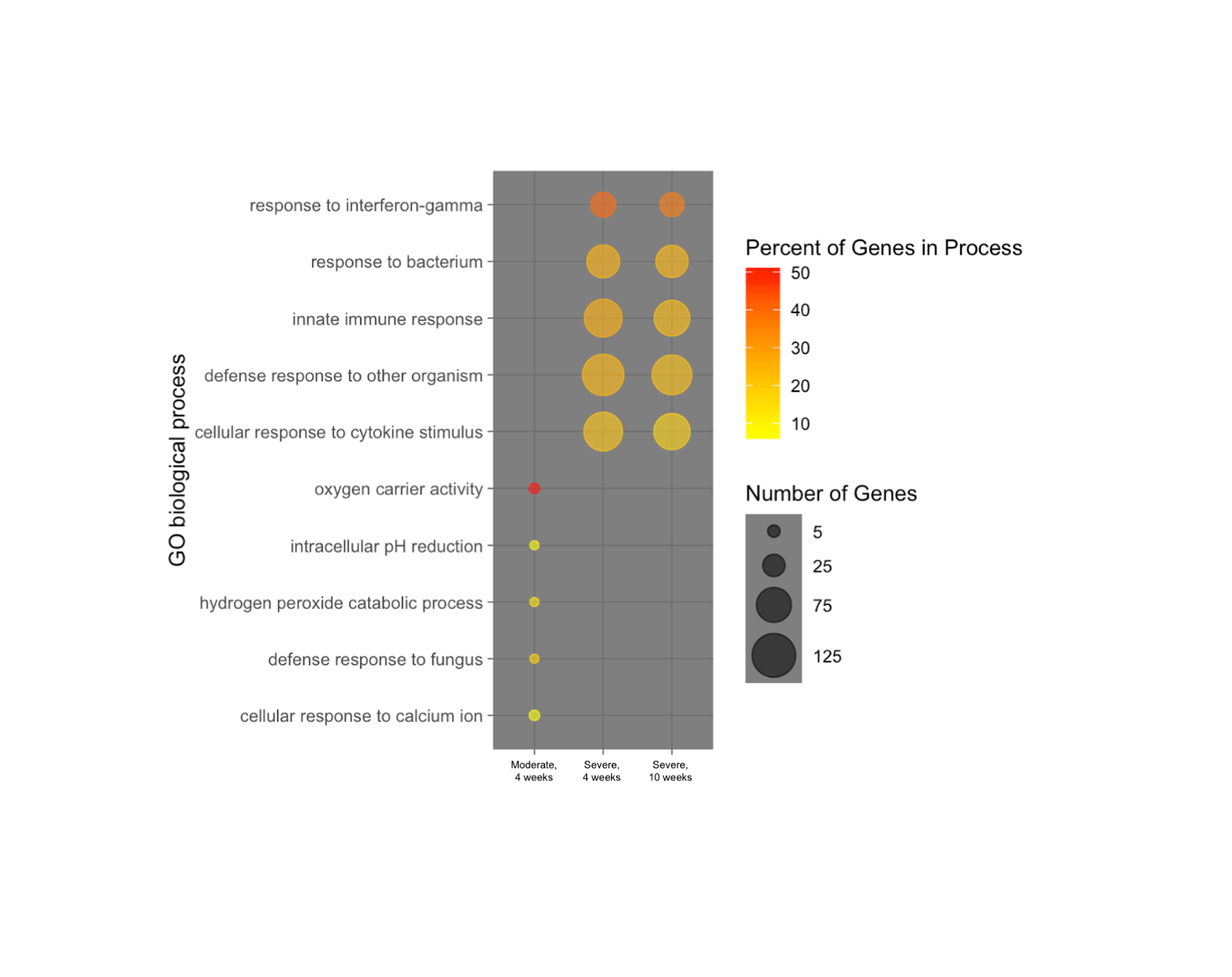

Supplement: S1 Fig — Top 5 enriched biological processes from each group based on lowest p values. P-values all < 0.05. Uninfected cattle n = 5, moderately affected cattle n = 3, severely affected cattle n = 4. (TIF) [file pone.0239938.s009.tif]

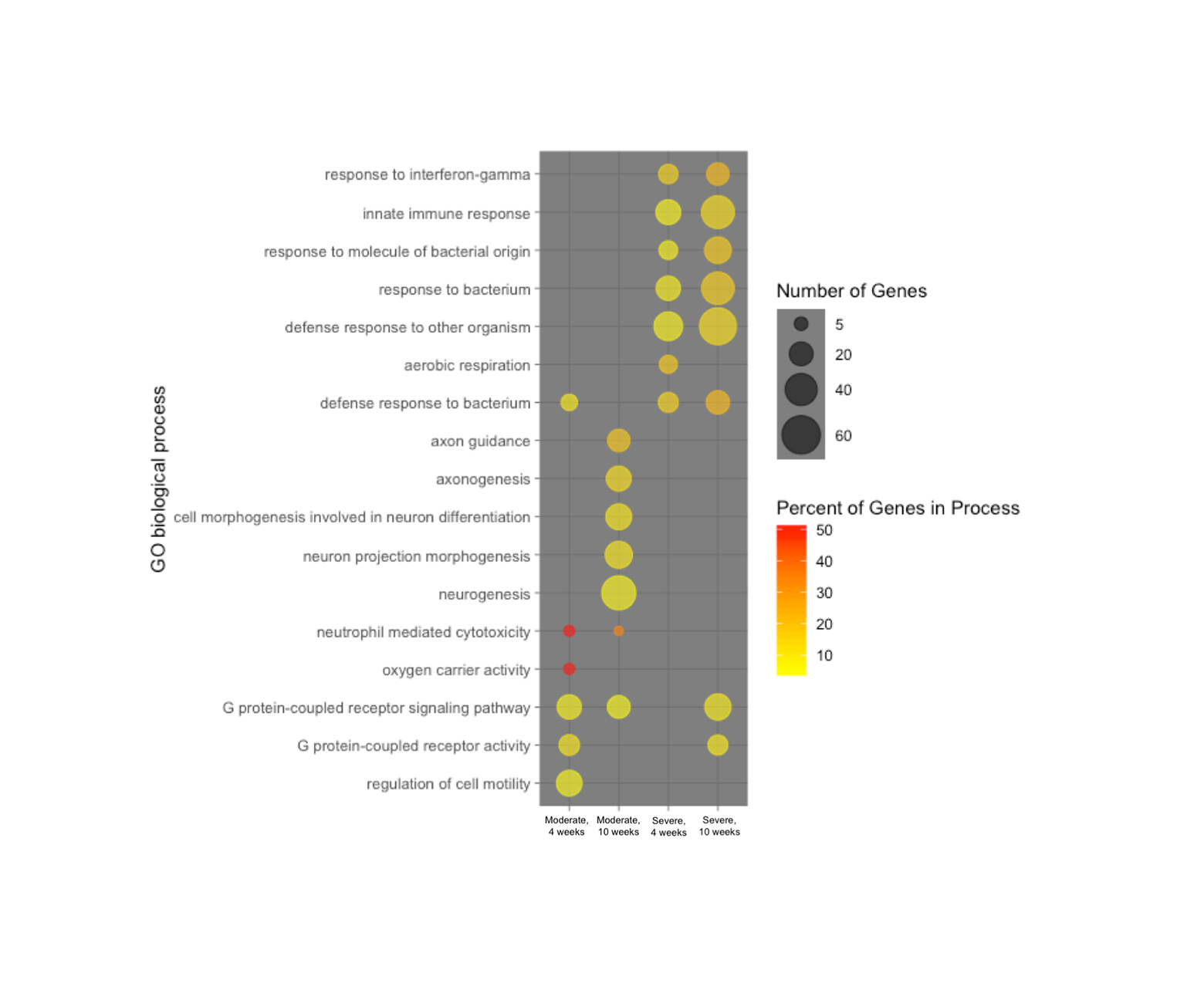

Supplement: S2 Fig — Top 5 enriched biological processes from each group based on lowest p values. P-values all < 0.05. Uninfected cattle n = 5, moderately affected cattle n = 3, severely affected cattle n = 4. (TIF) [file pone.0239938.s010.tif]

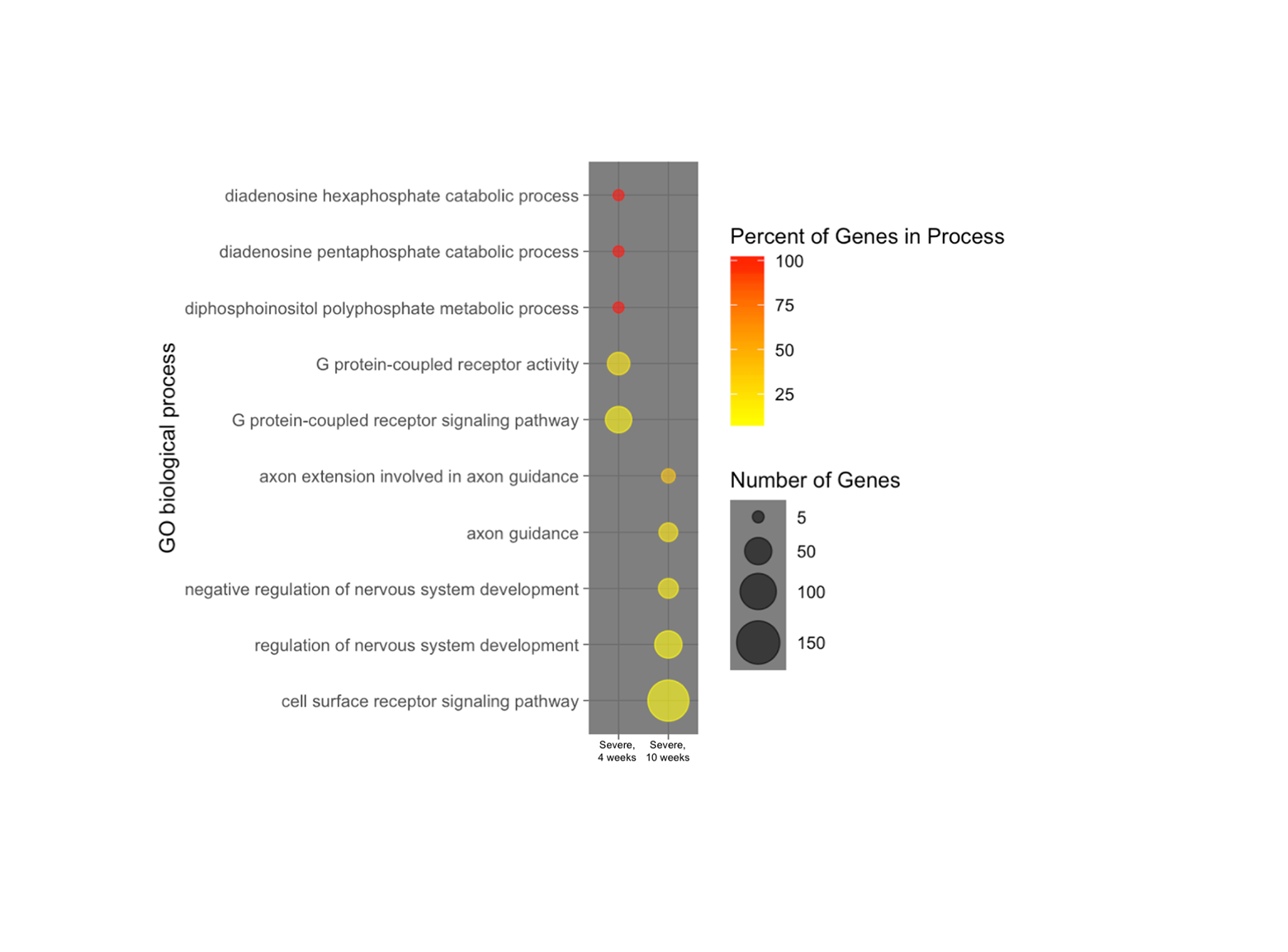

Supplement: S3 Fig — Top 5 enriched biological processes from each group based on lowest p values. P-values all < 0.05. Uninfected cattle n = 5, moderately affected cattle n = 3, severely affected cattle n = 4. (TIF) [file pone.0239938.s011.tif]
