# Supplementary material for: Severity of bovine tuberculosis is associated with innate immune-biased transcriptional signatures of whole blood in early weeks after experimental Mycobacterium bovis infection
Source: PLoS One. 2020 Nov 9;15(11):e0239938. doi: 10.1371/journal.pone.0239938 (PMC7652326; doi:10.1371/journal.pone.0239938)
Supplement: Attachment — Submitted filename: Response to Reviewers.docx [file pone.0239938.s013.docx]

Dear PLOS ONE Editor,

We thank you, again, for your review of the manuscript entitled “*Severity of bovine tuberculosis is associated with innate immune-biased transcriptional signatures of whole blood in early weeks after experimental* Mycobacterium bovis *infection*.” We have addressed the second round of comments suggested by the reviewer and hope that we have taken care of all concerns.

**Reviewer comments**

*The new Figure 1 is an excellent graphical representation of the work and make it much easier for readers to understand how the study was conducted.*

We hoped this figure would improve the manuscript and thank the reviewer for their comment.

*Line 54-55: This statement doesn't currently make any sense. The citation is from 1995 and the year being referred to 2004. Is there a citation for the $50 million cattle statistic? If so, the text could be re-worded as follows - note insertion of new citation and "at least" before "$3 billion"
"In 2004, it was estimated that worldwide, an estimated >50 million cattle are infected with M. bovis (CITATION), resulting in a financial loss of at least $3 billion USD annually (6)."*

We apologize for the confusion with this statement. The “2004” date is a mistake, so that has been corrected. We have not been able to find a more recent citation for the number of cattle and cost associated with bovine tuberculosis worldwide. While old, this is the citation that is still currently used by ourselves and others.

*Lines 258 and 259: Please make it clear you are using Log2FC values (with subscript "2"). "LogFC" is not sufficient - a non-expert reader will assume this is Log10.
Lines 275 and 276: Same as previous comment.*

We apologize for the oversight, this has been addressed.

Supplementary Files 3 and 4: The terms "upregulated" and "downregulated" are still being used inappropriately in these files. Please change to "increased expression" or "decreased expression" etc. as detailed in the review of the first version of the manuscript.

We apologize for the oversight, we have made these changes accordingly.

Again, we thank the editor and reviewers for their time and effort in reviewing our manuscript. We hope that we have addressed all concerns and hope that you will find this manuscript suitable for publication.

Thank you,

Paola M. Boggiatto
